# Diagnostic Value of Immunological Biomarkers in Children with Asthmatic Bronchitis and Asthma

**DOI:** 10.3390/medicina59101765

**Published:** 2023-10-03

**Authors:** Ming Wu, Danru Liu, Fenhua Zhu, Yeheng Yu, Zhicheng Ye, Jin Xu

**Affiliations:** Department of Clinical Laboratory, Children’s Hospital of Fudan University, National Children’s Medical Center, Shanghai 201102, China; wming@fudan.edu.cn (M.W.); danruliu1981@163.com (D.L.); 13641754437@163.com (F.Z.); 13816116755@163.com (Y.Y.); yzcrxj@163.com (Z.Y.)

**Keywords:** immunological biomarkers, machine learning, random forest, asthma, asthmatic bronchitis

## Abstract

*Background and Objectives*: This study aimed to investigate the diagnostic value of immunological biomarkers in children with asthmatic bronchitis and asthma and to develop a machine learning (ML) model for rapid differential diagnosis of these two diseases. *Materials and Methods*: Immunological biomarkers in peripheral blood were detected using flow cytometry and immunoturbidimetry. The importance of characteristic variables was ranked and screened using random forest and extra trees algorithms. Models were constructed and tested using the Scikit-learn ML library. K-fold cross-validation and Brier scores were used to evaluate and screen models. *Results*: Children with asthmatic bronchitis and asthma exhibit distinct degrees of immune dysregulation characterized by divergent patterns of humoral and cellular immune responses. CD8^+^ T cells and B cells were more dominant in differentiating the two diseases among many immunological biomarkers. Random forest showed a comprehensive high performance compared with other models in learning and training the dataset of immunological biomarkers. *Conclusions*: This study developed a prediction model for early differential diagnosis of asthmatic bronchitis and asthma using immunological biomarkers. Evaluation of the immune status of patients may provide additional clinical information for those children transforming from asthmatic bronchitis to asthma under recurrent attacks.

## 1. Introduction

Bronchitis is a prevalent respiratory disease in children that can be categorized into several types, such as plastic bronchitis, asthmatic bronchitis, obliterans bronchitis, and bronchiolitis. Asthmatic bronchitis is the most common type of bronchitis in children and shares some clinical symptoms with asthma [1]. These symptoms include coughing, wheezing with expiratory dyspnea, and night-time exacerbations [2]. Children with bronchial stenosis have weakened defense functions and immune systems, making them more susceptible to recurrent attacks of asthmatic bronchitis that can potentially progress to asthma [3]. Such progression increases the difficulty of treatment and poses a serious threat to the lives and health of affected children. Additionally, this process is typically irreversible.

An increasing number of innate and adaptive immune cell types and cytokines have been identified as critical drivers of asthmatic diseases, particularly asthma. The interaction between these factors is also a fundamental cause of disease development [4,5]. Understanding the immune status of children by detecting immunological biomarkers in peripheral blood has significant guiding implications for the differential diagnosis and treatment of asthma.

As a field of artificial intelligence (AI), the development of machine learning (ML) has brought dramatic changes to the medical field, including exponential increases in computing power, big data processing and mining technologies, and access to large clinical data sets using electronic health records [6]. ML focuses on developing algorithms to best represent a set of data. It involves probability theory, statistics, approximation theory, and complex algorithms. The core of ML is to learn from existing data sets, study the connections between data, and generate a new algorithm and train it to classify or predict related events [7].

In this study, we chose to focus on several key immunological biomarkers for assessment, including lymphocyte subsets, immunoglobulins, and complements. These biomarkers were selected based on their established association with immune responses and their potential role in the pathogenesis of asthmatic diseases. Variations in lymphocyte subsets can indicate alterations in immune responses, while changes in immunoglobulin levels can reflect the body’s ability to respond to allergens. The levels of complements are often associated with the overall state of the immune system, playing a crucial role in immune defense mechanisms. By analyzing these immunological biomarkers, this study aims to provide a more nuanced understanding of the immune statuses of children with asthmatic bronchitis and asthma. Furthermore, we endeavored to establish an immune prediction model to identify these two diseases, thereby assisting doctors in making accurate diagnoses and offering more clinical information to prevent the further development of asthmatic bronchitis into asthma.

## 2. Materials and Methods

### 2.1. Patients and Specimens

Children with asthmatic bronchitis and asthma diagnosed at the Children’s Hospital of Fudan University from June 2021 to June 2022 were enrolled as research subjects. A total of 61 children with asthmatic bronchitis and 72 children with asthma were enrolled in this study (Table 1). The following are the inclusion and exclusion criteria:

Patients with asthmatic bronchitis were diagnosed according to the criteria outlined in Zhu Futang’s Practical Pediatrics (8th Edition) [8]. These criteria include an acute onset characterized by a short disease course and rapid progression, often accompanied by symptoms of upper respiratory infection such as sore throat and nasal congestion. Patients frequently exhibit an irritative dry cough or expectorate a small amount of mucous sputum and may experience a sensation of chest tightness. When not coughing, sounds of phlegm and wheezing can often be heard in the throat, although there is no obvious difficulty in breathing. Cough and wheeze exacerbate during the night or early morning, resembling asthma, and severe wheezing can lead to cyanosis. Complete blood count tests often indicate abnormalities in white blood cells or neutrophils.

Patients with asthma met the diagnostic criteria of the Global Initiative for Asthma (GINA) guidelines [9,10,11], which encompass episodes of wheezing, breathlessness, chest tightness, or coughing, positive bronchial provocation test or exercise provocation test, positive bronchial dilation test with an increase in forced expiratory volume in one second (FEV1) of >12% and an absolute increase in FEV1 of ≥200 mL, and a variability rate of ≥20% in the peak expiratory flow (PEF) within a day (or within 2 weeks).

Exclusion criteria include patients with other respiratory diseases, autoimmune diseases, malignant tumors, severe heart, liver, or kidney function impairment, or those who have used systemic or inhaled corticosteroids or bronchodilators within two weeks before admission. A total of 4–5 mL of fasting venous blood was extracted into a sterile heparin sodium anticoagulant tube and serum separation gel-procoagulant tube.

### 2.2. Detection of Lymphocyte Subsets

Approximately 10 μL of fluorescent antibody was aspirated into a Trucount tube (FITC- CD3, clone SK7; PE-CD16 and CD56, clone B73.1 and NCAM16.2; PerCP-Cy 5.5-CD45, clone 2D1 (HLe-1); 21 PE-Cy7-CD4, clone SK3; APC-CD19, clone SJ25C1; APC-Cy7-CD8, clone SK1), then 50 μL of whole blood was subsequently added and incubated for 15 min. 450 μL of hemolysin were added in the dark at room temperature and allowed to remain at room temperature for 5 min to completely lysate erythrocytes. Fluorescence data were collected on BD FACS Calibur flow cytometry (BD Biosciences, San Jose, CA, USA) and analyzed with FlowJo software v10 (Tree Star, Ashland, OR, USA).

### 2.3. Detection of Immunoglobulin and Complement

The standard curve was drawn with the absorbance as the *y*-axis and the concentration of the calibration solution as the *x*-axis. Thereafter, 10 μL serum was added to 250 μL phosphate buffer and incubated at 37 °C for 3–5 min, and absorbance A1 at 600 nm was detected. Then 50 μL latex particles coated with rabbit anti-human antibody were added and incubated at 37 °C for 5 min, and absorbance A2 at 600 nm was detected. The corresponding concentration value was calculated according to ΔA (A2−A1) and the standard curve.

### 2.4. ML Tools and Process

The ML system developed for this study was written in Python programming language version 3.9.13 using the Scikit-learn ML library, and the code was drafted using Jupyter Notebook running the IPython kernel by visual studio code software (Microsoft Corporation, Redmond, WA, USA). The ML process in this research consisted of five steps: data preprocessing, feature ranking and screening, model building, testing, and visualization. The data preprocessing step included data missing filling, variable coding, and normalization. Seven algorithms were utilized in this study, namely support vector machine (SVM), logistic regression (LR), random forest (RF), extra trees (ET), decision tree (DT), Gaussian NB, and k-nearest neighbor (KNN). Models were constructed and tested using the Scikit-learn ML library. K-fold cross-validation and Brier scores were used to evaluate and screen models.

### 2.5. Statistical Analysis

SPSS 25.0 statistical software (IBM Corp., Armonk, NY, USA) was used for data analysis. The measurement data conforming to normal distribution were expressed as mean ± standard deviation (X ± S), and non-normally distributed measurement data were expressed as median (Interquartile range) (M (Q25, Q75), %). The differences between the two groups were compared using an independent samples *t*-test or the non-parametric Mann-Whitney U test. *p* < 0.05 was considered statistically significant.

## 3. Results

### 3.1. Comparison of Immunological Biomarkers in Children with Asthmatic Bronchitis and Asthma

**Figure 1:**
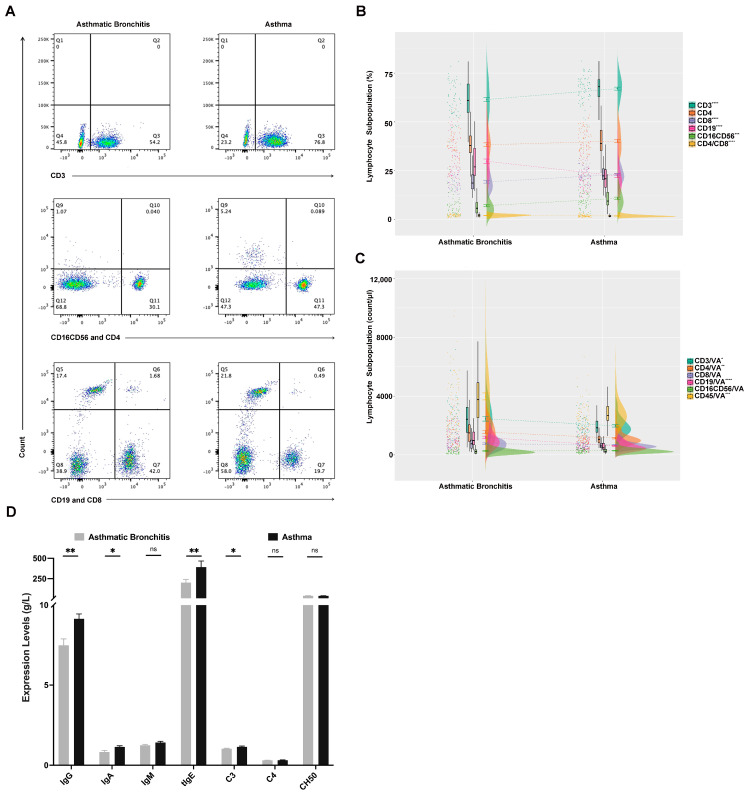
Lymphocyte subsets, immunoglobulin and complement levels in children with asthmatic bronchitis and asthma. (**A**) The representative flow cytometry analysis plots of lymphocyte subsets in children with asthmatic bronchitis and asthma (CD3^+^, CD4^+^, CD8^+^, CD16^+^CD56^+^, CD19^+^). (**B**) Percentage of lymphocyte subsets in children with asthmatic bronchitis and asthma. (**C**) Absolute count of lymphocyte subsets in children with asthmatic bronchitis and asthma. (**D**) Immunoglobulin and complement levels in children with asthmatic bronchitis and asthma. ^ns^ *p* > 0.05, * *p* < 0.05, ** *p* < 0.01, *** *p* < 0.001, **** *p* < 0.0001.

We first used flow cytometry to investigate the expression of lymphocyte subsets and a gating strategy to sort lymphocyte subsets according to different fluorescent dyes (Figure 1A). As depicted in Figure 1B, levels of CD3, CD4, and CD45 absolute count, CD4/CD8, and B cell (CD19 percentage and absolute count) were higher in the asthmatic bronchitis group than in the asthma groups, while levels of CD3, CD8, and CD16CD56 percentage were lower (*p* < 0.05). Furthermore, we detected serum immunoglobulin and complement levels in children with asthmatic bronchitis and asthma. As shown in Figure 1C, IgG, IgA, tIgE, and C3 levels were lower in the asthmatic bronchitis group than in the asthma children (*p* < 0.05).

### 3.2. Importance Ranking and Selection of Immunological Biomarkers

**Figure 2:**
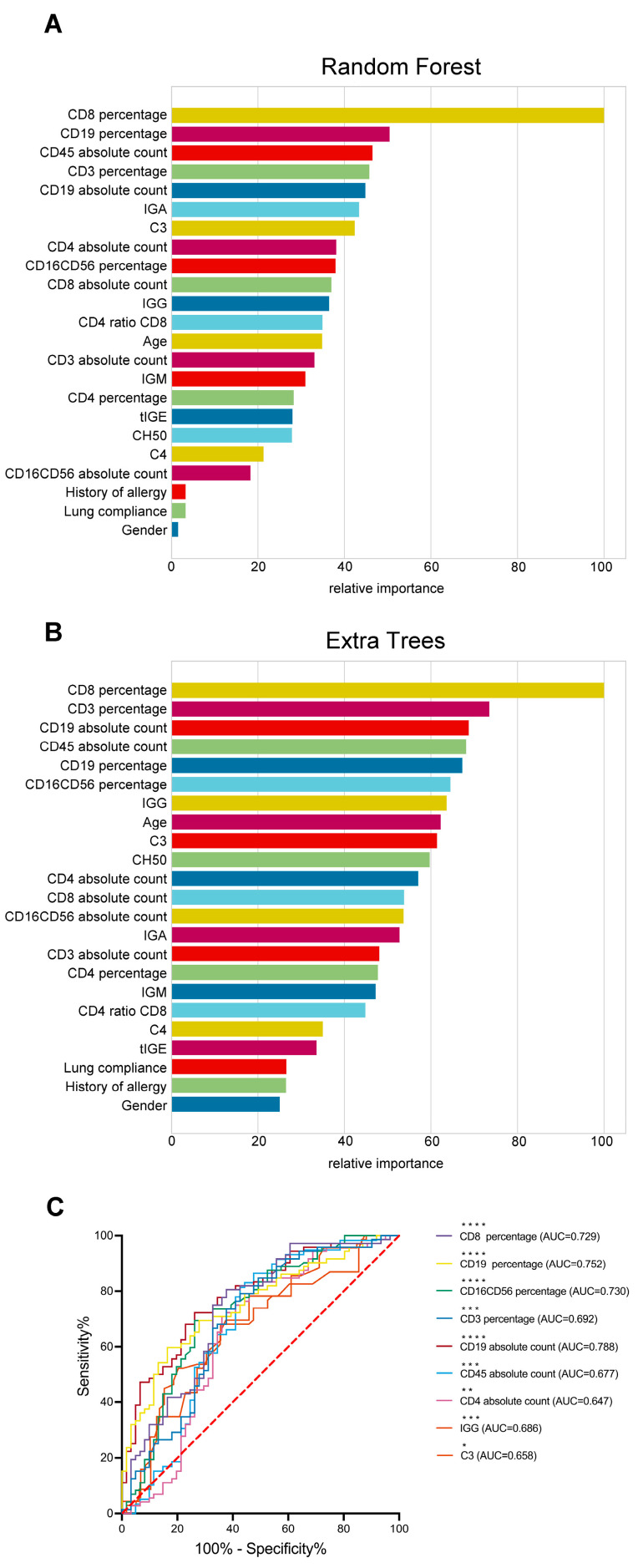
Importance ranking and selection of immunological biomarkers for differentiating asthmatic bronchitis from asthma. (**A**,**B**) Histogram of importance ranking results of immunological biomarkers by RF and ET classifier. (**C**) ROC curve of immunological biomarkers after screening to differentiate asthmatic bronchitis from asthma, * *p* < 0.05, ** *p* < 0.01, *** *p* < 0.001, **** *p* < 0.0001.

Due to the large variety of immunological biomarkers, we used RF and ET to rank and screen the importance of features (immunological biomarkers and demographic characteristics). As shown in Figure 2A,B, the importance of each feature in discriminating between asthma and asthmatic bronchitis is presented in descending order. Lymphocyte subsets, compared with immunoglobulins and complement, were more important for differentiating asthmatic bronchitis from asthma, especially CD19 percentage and absolute count, which rank high.

In addition, the characteristics obtained by RF and ET screening are as follows according to different integrated learning thought. RF: Age, CD3 percentage, CD8 percentage, CD19 percentage, CD16CD56 percentage, CD4 ratio CD8, CD4 absolute count, CD8 absolute count, CD19 absolute count, CD45 absolute count, IGG, IGA, C3; ET: Age, CD3 percentage, CD8 percentage, CD19 percentage, CD16CD56 percentage, CD4 absolute count, CD19 absolute count, CD45 absolute count, IGG, C3, CH50. The intersection of features screened by the two classifiers was selected for receiver operating characteristic (ROC) analysis (Figure 2C). Combined with the results of difference analysis, features without statistical difference were excluded, and a total of 10 features were finally obtained which were age, CD3 percentage, CD8 percentage, CD19 percentage, CD16CD56 percentage, CD4 absolute count, CD19 absolute count, CD45 absolute count, IGG, C3.

### 3.3. Model Building and Testing

To optimize the utilization of the data, the raw data was divided into two parts: the training dataset which accounted for approximately 70% of the original data, and the test dataset which accounted for the remaining 30%. The test set was not involved in any model building or preparation, ensuring that it truly represented new and unknown data. After importing the estimator from the Scikit-learn library, a new model was created and assigned to a variable named “model”. The fit() function was then utilized to train the model with the training dataset, enabling it to possess discriminative classification abilities. The predict function was subsequently employed to input the test dataset and evaluate the model’s credibility based on its training.

**Figure 3:**
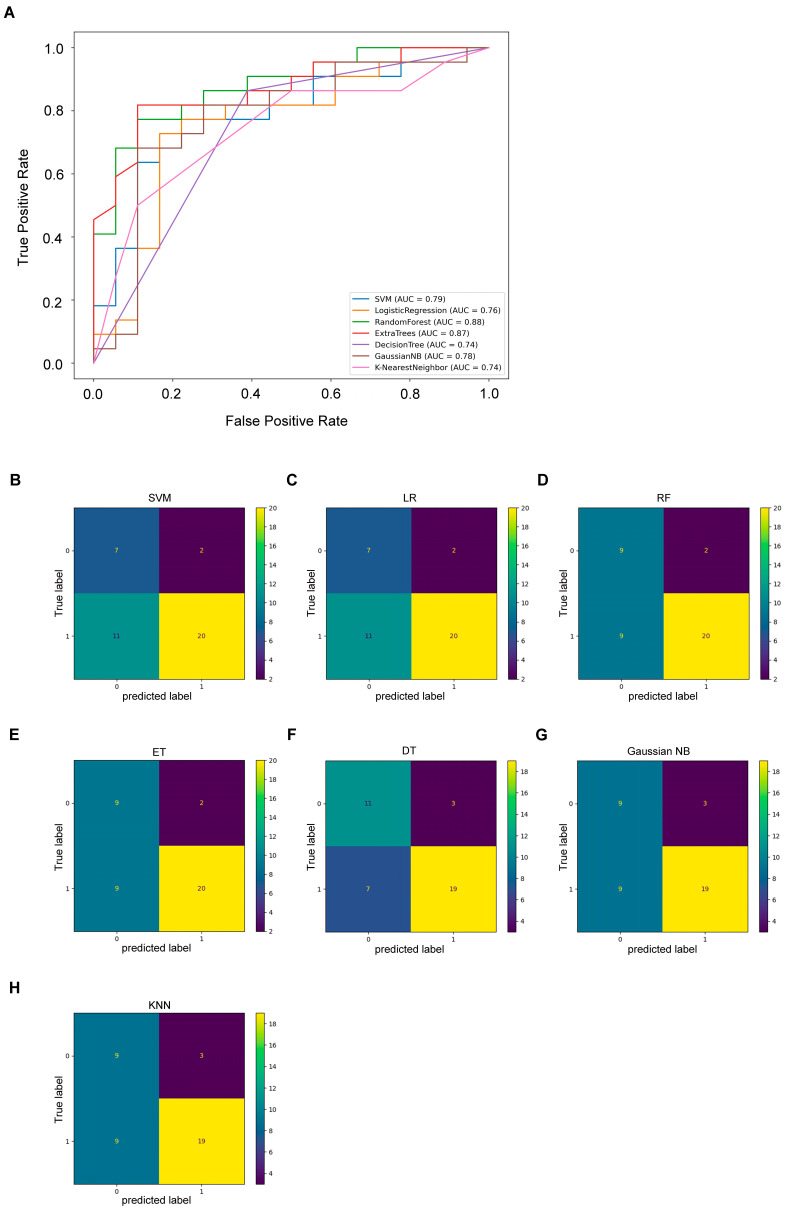
Model Testing. (**A**) ROC curve of seven models to differentiate asthmatic bronchitis from asthma. (**B–H**) Confusion matrix of seven models to differentiate asthmatic bronchitis from asthma. SVM = Support Vector Machine, LR = Logistic Regression, RF = Random Forest, ET = Extra Trees; DT = Decision Tree; KNN = K-Nearest Neighbor.

Confusion matrix and ROC were used to analyze the ability of the model to identify these two diseases (Figure 3A–H) and results are presented in Table 2, with SVM achieving an area under the curve (AUC) of 0.79 and accuracy of 0.675, LR achieving an AUC of 0.76 and accuracy of 0.675, RF achieving an AUC of 0.88 and accuracy of 0.725, ET achieving an AUC of 0.87 and accuracy of 0.725, DT achieving an AUC of 0.74 and accuracy of 0.75, Gaussian NB achieving an AUC of 0.78 and accuracy of 0.7, and KNN achieving an AUC of 0.74 and accuracy of 0.7. Among the seven models analyzed, all demonstrated a substantial ability to differentiate between asthmatic bronchitis and asthma. Specifically, the DT model exhibited the highest accuracy, while the RF model achieved the highest AUC.

### 3.4. Model Building and Testing

The seven above-shown models demonstrated strong prediction performance, however, due to our small and limited dataset, overfitting is a concern. To further build a reliable and stable model, we evaluated above models using Brier Score and K-fold cross-validation. The Brier Score measures prediction and calibration quality on a scale of 0 to 1, with lower scores indicating better performance [12]. K-fold cross-validation evaluates model performance by calculating the average test error, reducing model variance with O(1/k) efficiency, and improving generalization ability [13,14].

**Figure 4:**
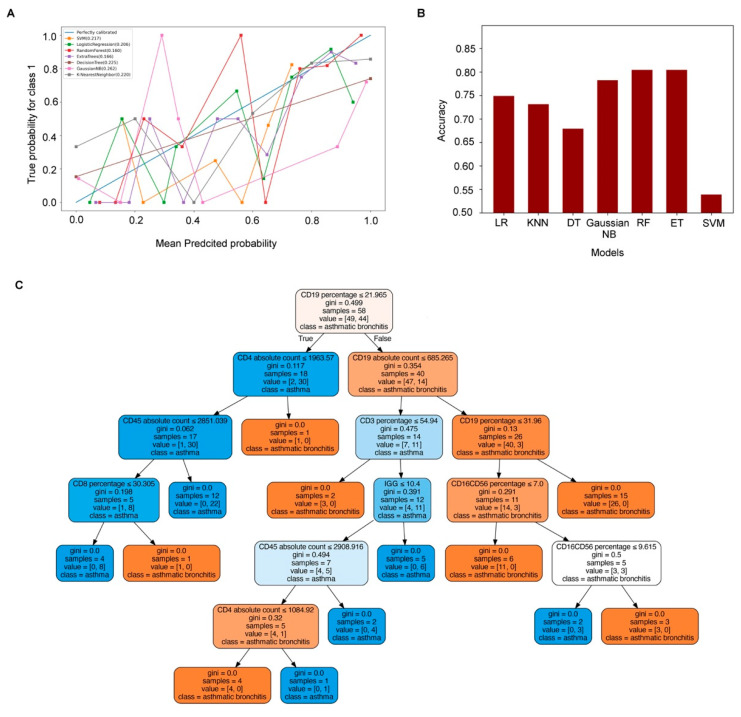
**Model screening and visualization.** (**A**) Reliability curve of seven models for differentiating asthmatic bronchitis from asthma. Brier Score = ∑(pi−oi)/N, pi = probability, oi = true results of samples (0 or 1). (**B**) K-fold cross-validation results of seven models. (**C**) Visualization of one of the decision trees in the RF model. Each leaf node has a different class (asthmatic bronchitis or asthma), which indicates which category will be divided according to the above rules. At the same time, each leaf node also has a value that indicates how many samples of each category are in this node.

Figure 4A,B shows that Brier Score of 0.217, 0.206, 0.160, 0.166, 0.225, 0.262, and 0.220 for SVM, LR, RF, ET, DT, Gaussian NB, and KNN models in the reliability curve, respectively. K-fold cross-validation yielded accuracy scores of 0.538889, 0.748889, 0.804444, 0.804444, 0.678889, 0.782222, and 0.731111, respectively. With the lowest Brier Score and highest accuracy obtained by RF, it was chosen for differentiating asthmatic bronchitis and asthma, which was also supported by the ROC analysis results.

RF is composed of multiple decision trees which is meaningful only to vote out the final output based on the results of all trees. There is no direct way to display RF when using Scikit-learn, which can only be ed into a single tree. Figure 4C visualizes one of the decision trees in RF; it started from the top of the tree, going down in turn according to whether the conditions were established or not, until the terminal node was reached, and the classification ended.

## 4. Discussion

Asthmatic bronchitis and asthma are heterogeneous diseases that require individualized and specific treatment [15,16]. Studying the mechanism of disease can provide effective information for clinical doctors to improve treatment. The change in lymphocyte subsets, immunoglobulin and complement levels in peripheral blood can reflect the status of the immune function of the body under different conditions [17,18]. In peripheral blood, it was found that B cells and CD4/CD8 were higher in the asthmatic bronchitis group than that in the asthma children, while levels of CD8^+^ T cells, and NK cells were lower. Furthermore, IgG, IgA, tIgE, and C3 levels in serum were lower than those in the asthma group. These results indicate that children with asthmatic bronchitis and asthma exhibit distinct degrees of immune dysregulation, characterized by divergent patterns of humoral and cellular immune responses.

Based on significance testing of difference, we also have performed an importance ranking of immunological biomarkers, and CD8^+^ T cells and B cells have shown high rankings in this assessment. CD8^+^ T lymphocytes belong to suppressor/killer T lymphocytes, and their main function is to kill target cells directly [19]. Compared to CD4^+^ T cells, there has been less research on CD8^+^ T cells in children with asthma. Both CD4^+^ T cells and CD8^+^ T cells can mediate hypersensitivity, but CD4^+^ (class II MHC molecules) and CD8^+^ T cells (class I MHC molecules) pertain to the recognition of antigens presented by different MHC molecules [20]. Some scholars have pointed out that compared to CD4^+^ T cells, CD8^+^ T cells have a stronger association with the severity of asthma [21]. CD8^+^ T cells that produce high levels of IFN-gamma (TC1 cells) have been shown to be associated with an attenuation of pulmonary allergic inflammation in rodent models [22]. In addition, levels of mononuclear cells, CD8^+^ T cells, and macrophages in bronchoalveolar lavage fluid of asthma patients are significantly elevated [23]. B cells can differentiate into plasma cells under antigen stimulation, then synthesize, store, and secrete antibodies (immunoglobulins) to participate in humoral immunity responses. B cells also have been recognized as important mediators in allergy and tolerance [24]. IgE secreted by B cells is a central player in childhood allergic reactions and one of the causes of wheezing [25]. The binding of IgE to its receptor on the surface of mast cells activates IgE-sensitized antigen-presenting cells (APCs) and Th2 cells, thereby promoting the production of IgE by B cells to supplement the IgE consumed in allergic reactions, and maintaining mast cells and APCs sensitization, causing bronchospasm or wheezing [26,27,28]. These studies suggest that both CD8^+^ T cells and B cells play important roles in the development and progression of both asthmatic bronchitis and asthma, which were consistent with our results.

Significance testing of difference is a type of hypothesis testing used to detect differences between experimental and control groups and determine their significance. Although the purpose is to discover more valuable information, it cannot achieve disease classification and assist in diagnosis [29,30]. ML can improve the accuracy and predictive ability of models by utilizing large amounts of data and fast-computing power, thereby helping doctors diagnose diseases and develop personalized treatment plans more accurately [31,32].

We established 7 ML models by using selected immunological biomarkers to distinguish between two types of diseases to help rapid diagnosis. However, different ML models have their own advantages and disadvantages, leading to varying results in model testing. Compared to other models, RF and ET performed better, with consistent accuracy in K-fold cross-validation and ML testing. RF and ET are similar in structure consisting of numerous decision trees, but they differ in the training set for each decision tree. RF uses the Bagging model (Bootstrap Aggregating) to randomly extract training samples for the training set, while ET uses all training samples [33]. After selecting the partition feature, the decision tree of RF will choose an optimal feature value partition point based on principles such as entropy, Gini index, and standard deviation, which is the same as traditional decision trees [34]. However, ET is more aggressive and randomly selects a feature value to partition the decision tree. Because the partition point of the feature value is randomly selected instead of the optimal point, this will generally result in a larger decision tree size than the decision tree generated by RF [35]. In other words, the variance of ET is further reduced relative to RF, but the bias is further increased. Considering the higher Brier score of ET in comparison to the RF model, the RF model was ultimately selected as the preferred choice.

Looking ahead, we envisage the integration of our model into telemedicine platforms, allowing for the remote monitoring and assessment of patients, thereby broadening the reach of healthcare services, especially in regions with limited access to healthcare facilities. This model could also foster advancements in research, aiding in the deeper exploration of the underlying mechanisms of these diseases, and potentially spearheading the development of novel therapeutic approaches. Moreover, it could serve as an educational tool, assisting in the training of healthcare professionals to recognize and comprehend the complex immunological patterns associated with these conditions. In the broader context, the insights garnered from the utilization of this model could influence policy and healthcare planning, shaping strategies aimed at alleviating the burden these diseases impose on the healthcare system.

However, our research also has several limitations that need to be addressed. The ML dataset was relatively small and lacked supplementary data such as radiological images, which might introduce bias into the results. Despite these current limitations, we plan to augment our dataset with a larger patient pool and incorporate multi-center studies to enhance the model’s accuracy in the future. This expansion would potentially allow for a more comprehensive diagnostic platform that integrates various diagnostic tools, offering a holistic view of the patient’s health status and fostering a more nuanced understanding of these diseases.

## 5. Conclusions

In conclusion, results of immunological biomarker detection suggest that children with asthma bronchitis and asthma exhibit varying degrees of immune dysfunction or disorder, which is linked to the type of disease. The detection of immunological biomarkers, particularly CD8^+^ T cells and B cells, followed by analysis using our model, can provide valuable assistance to clinicians in rapidly diagnosing the disease and obtaining a comprehensive understanding of the child’s immune status and disease progression. Ultimately, this approach may play an important role in preventing recurrent episodes of asthmatic bronchitis from progressing into asthma.

## Figures and Tables

**Table 1:** Characteristic features of children with asthmatic bronchitis and asthma (n, %).

Variables	Asthmatic Bronchitis (*n* = 61)	Asthma (*n* = 72)
*Gender*		
Male	49 (80.3)	47 (65.3)
Female	12 (19.7)	25 (34.7)
*Age (year)*		
≤1	11 (18)	6 (8.3)
1< and ≤2	22 (36.1)	13 (18.1)
>2	28 (45.9)	53 (73.6)
*History of allergy*		
Yes	27 (44.3)	52 (72.2)
No	34 (55.7)	20 (27.7)
*Lung compliance*		
Yes	9 (14.8)	17 (23.6)
No	52 (85.2)	55 (76.4)

**Table 2:** Performance of the models.

Models	Asthmatic Bronchitis			Asthma			Accuracy
	Precision	Recall	F1-Score	Precision	Recall	F1-Score	
SVM	0.39	0.78	0.52	0.91	0.65	0.75	0.675
LR	0.39	0.78	0.52	0.91	0.65	0.75	0.675
RF	0.5	0.82	0.62	0.91	0.69	0.78	0.725
ET	0.5	0.82	0.62	0.91	0.69	0.78	0.725
DT	0.61	0.79	0.69	0.86	0.73	0.79	0.75
Gaussian NB	0.5	0.75	0.6	0.86	0.68	0.76	0.7
KNN	0.5	0.75	0.6	0.86	0.68	0.76	0.7

Accuracy = (TP + TN)/(TP + TN + FP + FN), Precision = TP/(TP + FP), Recall = TP/(TP + FN), F1-score = 2TP/(2TP + FP + FN). TP: True Positive, FP: False Positive, TN: True Negative, FN: False Negative. SVM = Support Vector Machine, LR = Logistic Regression, RF = Random Forest, ET = Extra Trees; DT = Decision Tree; KNN = K-Nearest Neighbor.

## Data Availability

Not applicable.
